# (*E*)-*N*′-[4-(Dimethyl­amino)­benzyl­idene]-4-methyl­benzohydrazide methanol monosolvate

**DOI:** 10.1107/S1600536811029394

**Published:** 2011-07-23

**Authors:** Huanyu Liu, Yanchun Cai, Jianyong Wu, Zhuolin Li, Guanwen Li

**Affiliations:** a School of Chemistry and Chemical Engineering, Guangdong Pharmaceutical University, Zhongshan 528453, People’s Republic of China

## Abstract

In the title compound, C_17_H_19_N_3_O·CH_3_OH, the hydrazone mol­ecule exists in a *trans* geometry with respect to the methyl­idene unit and the dihedral angle between the two substituted benzene rings is 42.6 (2)°. In the crystal, the components are linked through N—H⋯O and O—H⋯O hydrogen bonds, forming [100] chains of alternating hydrazone and methanol mol­ecules.

## Related literature

For the hydrazone compounds reported by one of the authors recently and background refereences, see: Liu (2010*a* ▶,*b* ▶).
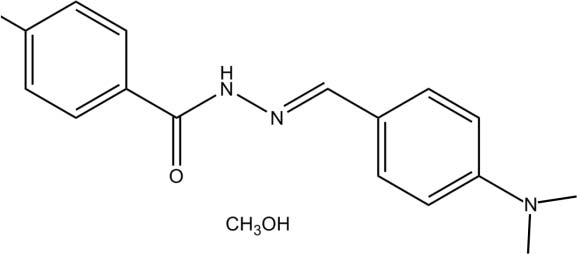


## Experimental

### 

#### Crystal data

C_17_H_19_N_3_O·CH_4_O
*M* *_r_* = 313.39
Triclinic, 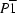

*a* = 6.3874 (18) Å
*b* = 11.724 (3) Å
*c* = 11.975 (3) Å
α = 78.830 (3)°
β = 77.138 (3)°
γ = 84.807 (3)°
*V* = 856.7 (4) Å^3^
*Z* = 2
Mo *K*α radiation
μ = 0.08 mm^−1^
*T* = 298 K
0.20 × 0.18 × 0.13 mm

#### Data collection

Bruker SMART CCD diffractometer
Absorption correction: multi-scan (*SADABS*; Bruker, 1998 ▶) *T*_min_ = 0.984, *T*_max_ = 0.990
5975 measured reflections
3613 independent reflections
1755 reflections with *I* > 2σ(*I*)
*R*_int_ = 0.030

#### Refinement

*R*[*F* ^2^ > 2σ(*F* ^2^)] = 0.060
*wR*(*F* ^2^) = 0.169
*S* = 1.00
3613 reflections
216 parameters
1 restraint
H atoms treated by a mixture of independent and constrained refinement
Δρ_max_ = 0.16 e Å^−3^
Δρ_min_ = −0.19 e Å^−3^

### 

Data collection: *SMART* (Bruker, 1998 ▶); cell refinement: *SAINT* (Bruker, 1998 ▶); data reduction: *SAINT*; program(s) used to solve structure: *SHELXS97* (Sheldrick, 2008 ▶); program(s) used to refine structure: *SHELXL97* (Sheldrick, 2008 ▶); molecular graphics: *SHELXTL* (Sheldrick, 2008 ▶); software used to prepare material for publication: *SHELXTL*.

## Supplementary Material

Crystal structure: contains datablock(s) global, I. DOI: 10.1107/S1600536811029394/hb6324sup1.cif

Structure factors: contains datablock(s) I. DOI: 10.1107/S1600536811029394/hb6324Isup2.hkl

Supplementary material file. DOI: 10.1107/S1600536811029394/hb6324Isup3.cml

Additional supplementary materials:  crystallographic information; 3D view; checkCIF report

## Figures and Tables

**Table 1:** Hydrogen-bond geometry (Å, °)

*D*—H⋯*A*	*D*—H	H⋯*A*	*D*⋯*A*	*D*—H⋯*A*
N2—H2*B*⋯O2^i^	0.90 (1)	2.02 (1)	2.905 (3)	168 (2)
O2—H2⋯O1	0.82	1.92	2.720 (3)	166

Symmetry code: (i) 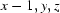.

**Fig. 1.**
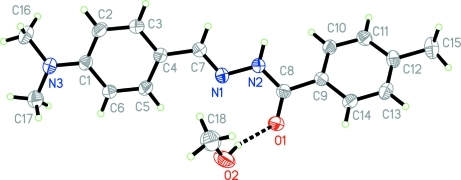
Molecular structure of the title compound with 30% probability displacement ellipsoids. Hydrogen bonds are shown as dashed lines.

**Fig. 2.**
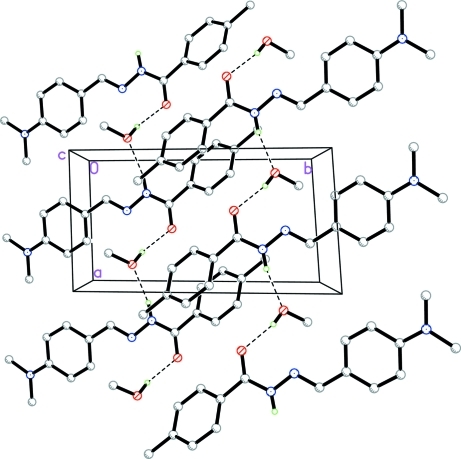
Packing structure of the title compound, viewed along the c axis. Hydrogen bonds are shown as dashed lines. Hydrogen atoms not involved in hydrogen bonding have been omitted.

## supplementary crystallographic information

### Comment

Recently, the author has reported two new hydrazone compounds (Liu, 2010*a*,*b*). As a further study on these compounds, in the present work, the title new hydrazone compound, (I), which crystallised as a methanol solvate, is reported.

In the title compound (Fig. 1), the methanol molecule is linked to the hydrazone molecule through O—H···O and O—H···N hydrogen bonds (Table 1). The hydrazone molecule exists in a *trans* geometry with respect to the methylidene unit. The dihedral angle between the two substituted benzene rings is 42.6 (2)°. In the crystal structure, the hydrazone and the methanol molecules are linked through N—H···O and O—H···O, hydrogen bonds (Table 1), to form chains along the *a* axis (Fig. 2).

### Experimental

4-Dimethylaminobenzaldehyde (1.0 mmol, 153 mg) and 4-methylbenzohydrazide (1.0 mmol, 150 mg) were mixed in 50 mL methanol. The mixture was stirred at ambient temperature for 2 h and filtered. Colorless blocks of the title compound were formed by slow evaporation of the filtrate for a week.

### Refinement

The amino hydrogen atom was located in an electronic density map and refined isotropically, with the N—H distance restrained to 0.90 (1)Å. Other hydrogen atoms were placed in calculated positions, with C—H = 0.93–0.96 Å, O—H = 0.82 Å, and refined as riding with U_iso_(H) = 1.2U_eq_(C) and 1.5U_eq_(O and methyl C).

### Crystal data

**Table:**

C_17_H_19_N_3_O·CH_4_O	*Z* = 2
*M**_r_* = 313.39	*F*(000) = 336
Triclinic, *P*1	*D*_x_ = 1.215 Mg m^−^^3^
*a* = 6.3874 (18) Å	Mo *K*α radiation, λ = 0.71073 Å
*b* = 11.724 (3) Å	Cell parameters from 744 reflections
*c* = 11.975 (3) Å	θ = 2.6–24.9°
α = 78.830 (3)°	µ = 0.08 mm^−^^1^
β = 77.138 (3)°	*T* = 298 K
γ = 84.807 (3)°	Block, colorless
*V* = 856.7 (4) Å^3^	0.20 × 0.18 × 0.13 mm

### Data collection

**Table:**

Bruker SMART CCD diffractometer	3613 independent reflections
Radiation source: fine-focus sealed tube	1755 reflections with *I* > 2σ(*I*)
graphite	*R*_int_ = 0.030
ω scans	θ_max_ = 27.0°, θ_min_ = 2.7°
Absorption correction: multi-scan (*SADABS*; Bruker, 1998)	*h* = −8→8
*T*_min_ = 0.984, *T*_max_ = 0.990	*k* = −14→13
5975 measured reflections	*l* = −15→15

### Refinement

**Table:**

Refinement on *F*^2^	Primary atom site location: structure-invariant direct methods
Least-squares matrix: full	Secondary atom site location: difference Fourier map
*R*[*F*^2^ > 2σ(*F*^2^)] = 0.060	Hydrogen site location: inferred from neighbouring sites
*wR*(*F*^2^) = 0.169	H atoms treated by a mixture of independent and constrained refinement
*S* = 1.00	*w* = 1/[σ^2^(*F*_o_^2^) + (0.0667*P*)^2^] where *P* = (*F*_o_^2^ + 2*F*_c_^2^)/3
3613 reflections	(Δ/σ)_max_ < 0.001
216 parameters	Δρ_max_ = 0.16 e Å^−^^3^
1 restraint	Δρ_min_ = −0.19 e Å^−^^3^

### Special details

**Table:**

Geometry. All esds (except the esd in the dihedral angle between two l.s. planes) are estimated using the full covariance matrix. The cell esds are taken into account individually in the estimation of esds in distances, angles and torsion angles; correlations between esds in cell parameters are only used when they are defined by crystal symmetry. An approximate (isotropic) treatment of cell esds is used for estimating esds involving l.s. planes.
Refinement. Refinement of F^2^ against ALL reflections. The weighted R-factor wR and goodness of fit S are based on F^2^, conventional R-factors R are based on F, with F set to zero for negative F^2^. The threshold expression of F^2^ > 2sigma(F^2^) is used only for calculating R-factors(gt) etc. and is not relevant to the choice of reflections for refinement. R-factors based on F^2^ are statistically about twice as large as those based on F, and R- factors based on ALL data will be even larger.

### Fractional atomic coordinates and isotropic or equivalent isotropic displacement parameters (Å^2^)

**Table:**

	*x*	*y*	*z*	*U*_iso_*/*U*_eq_	
N1	0.4251 (3)	0.17760 (17)	0.23171 (17)	0.0519 (6)	
N2	0.2884 (3)	0.26788 (18)	0.27010 (18)	0.0518 (5)	
N3	0.7692 (4)	−0.29356 (18)	0.03356 (18)	0.0603 (6)	
O1	0.5681 (3)	0.37103 (15)	0.27273 (17)	0.0699 (6)	
O2	0.8472 (3)	0.19729 (18)	0.3439 (2)	0.0945 (7)	
H2	0.7518	0.2406	0.3205	0.142*	
C1	0.6665 (4)	−0.2025 (2)	0.0848 (2)	0.0493 (6)	
C2	0.4491 (4)	−0.2032 (2)	0.1361 (2)	0.0584 (7)	
H2A	0.3721	−0.2677	0.1391	0.070*	
C3	0.3463 (4)	−0.1095 (2)	0.1824 (2)	0.0595 (7)	
H3	0.2001	−0.1121	0.2154	0.071*	
C4	0.4524 (4)	−0.0118 (2)	0.1816 (2)	0.0498 (6)	
C5	0.6711 (4)	−0.0127 (2)	0.1337 (2)	0.0563 (7)	
H5	0.7481	0.0511	0.1331	0.068*	
C6	0.7761 (4)	−0.1051 (2)	0.0874 (2)	0.0557 (7)	
H6	0.9233	−0.1033	0.0571	0.067*	
C7	0.3337 (4)	0.0872 (2)	0.2259 (2)	0.0547 (7)	
H7	0.1853	0.0847	0.2511	0.066*	
C8	0.3731 (4)	0.3636 (2)	0.2844 (2)	0.0494 (6)	
C9	0.2198 (4)	0.4615 (2)	0.31429 (19)	0.0450 (6)	
C10	0.0261 (4)	0.4838 (2)	0.2788 (2)	0.0518 (7)	
H10	−0.0156	0.4341	0.2368	0.062*	
C11	−0.1058 (4)	0.5789 (2)	0.3052 (2)	0.0566 (7)	
H11	−0.2332	0.5937	0.2783	0.068*	
C12	−0.0529 (5)	0.6528 (2)	0.3707 (2)	0.0541 (7)	
C13	0.1388 (5)	0.6292 (2)	0.4075 (2)	0.0601 (7)	
H13	0.1772	0.6769	0.4525	0.072*	
C14	0.2749 (4)	0.5360 (2)	0.3787 (2)	0.0534 (7)	
H14	0.4051	0.5231	0.4029	0.064*	
C15	−0.2024 (5)	0.7539 (2)	0.4031 (2)	0.0797 (9)	
H15A	−0.1317	0.8013	0.4390	0.119*	
H15B	−0.3298	0.7250	0.4567	0.119*	
H15C	−0.2408	0.7996	0.3343	0.119*	
C16	0.6496 (5)	−0.3900 (2)	0.0267 (2)	0.0713 (8)	
H16A	0.6002	−0.4333	0.1035	0.107*	
H16B	0.7407	−0.4398	−0.0206	0.107*	
H16C	0.5283	−0.3608	−0.0073	0.107*	
C17	0.9965 (5)	−0.2948 (2)	−0.0162 (3)	0.0775 (9)	
H17A	1.0262	−0.2307	−0.0798	0.116*	
H17B	1.0406	−0.3666	−0.0439	0.116*	
H17C	1.0740	−0.2879	0.0421	0.116*	
C18	0.7566 (5)	0.1029 (3)	0.4231 (3)	0.0921 (11)	
H18A	0.8683	0.0469	0.4421	0.138*	
H18B	0.6613	0.0677	0.3895	0.138*	
H18C	0.6774	0.1288	0.4925	0.138*	
H2B	0.1469 (18)	0.257 (2)	0.292 (2)	0.080*	

### Atomic displacement parameters (Å^2^)

**Table:**

	*U*^11^	*U*^22^	*U*^33^	*U*^12^	*U*^13^	*U*^23^
N1	0.0519 (13)	0.0437 (12)	0.0603 (14)	0.0045 (11)	−0.0138 (10)	−0.0106 (10)
N2	0.0470 (13)	0.0442 (12)	0.0652 (14)	0.0003 (11)	−0.0110 (11)	−0.0142 (10)
N3	0.0649 (16)	0.0474 (13)	0.0717 (15)	−0.0017 (12)	−0.0149 (12)	−0.0181 (11)
O1	0.0476 (12)	0.0561 (12)	0.1090 (16)	−0.0009 (9)	−0.0189 (10)	−0.0201 (10)
O2	0.0519 (13)	0.0742 (15)	0.147 (2)	0.0031 (11)	−0.0245 (13)	0.0062 (14)
C1	0.0572 (17)	0.0415 (14)	0.0500 (15)	−0.0037 (13)	−0.0151 (12)	−0.0048 (12)
C2	0.0613 (18)	0.0485 (16)	0.0673 (18)	−0.0154 (14)	−0.0106 (14)	−0.0129 (13)
C3	0.0467 (16)	0.0586 (17)	0.0754 (19)	−0.0063 (13)	−0.0093 (13)	−0.0198 (15)
C4	0.0513 (16)	0.0435 (15)	0.0556 (16)	−0.0021 (12)	−0.0122 (12)	−0.0100 (12)
C5	0.0564 (18)	0.0445 (15)	0.0668 (18)	−0.0111 (13)	−0.0067 (14)	−0.0102 (13)
C6	0.0454 (15)	0.0507 (16)	0.0707 (18)	−0.0079 (13)	−0.0059 (13)	−0.0148 (14)
C7	0.0490 (16)	0.0510 (16)	0.0647 (18)	−0.0015 (13)	−0.0106 (13)	−0.0137 (13)
C8	0.0491 (16)	0.0465 (15)	0.0519 (15)	−0.0034 (13)	−0.0116 (12)	−0.0057 (12)
C9	0.0453 (15)	0.0428 (14)	0.0447 (14)	−0.0027 (12)	−0.0084 (11)	−0.0037 (11)
C10	0.0550 (17)	0.0513 (16)	0.0525 (15)	−0.0004 (13)	−0.0147 (13)	−0.0142 (12)
C11	0.0539 (17)	0.0612 (17)	0.0537 (16)	0.0036 (14)	−0.0137 (13)	−0.0080 (13)
C12	0.0670 (19)	0.0428 (15)	0.0464 (15)	0.0046 (13)	−0.0049 (13)	−0.0044 (12)
C13	0.076 (2)	0.0510 (16)	0.0545 (17)	−0.0121 (15)	−0.0065 (14)	−0.0174 (13)
C14	0.0561 (16)	0.0490 (16)	0.0579 (16)	−0.0079 (13)	−0.0166 (13)	−0.0088 (13)
C15	0.101 (3)	0.0611 (19)	0.0695 (19)	0.0140 (17)	−0.0032 (17)	−0.0179 (15)
C16	0.091 (2)	0.0502 (17)	0.076 (2)	−0.0128 (16)	−0.0134 (16)	−0.0202 (14)
C17	0.073 (2)	0.073 (2)	0.088 (2)	−0.0007 (17)	−0.0077 (17)	−0.0311 (17)
C18	0.090 (3)	0.074 (2)	0.112 (3)	0.004 (2)	−0.035 (2)	−0.005 (2)

### Geometric parameters (Å, °)

**Table:**

N1—C7	1.275 (3)	C9—C10	1.383 (3)
N1—N2	1.392 (3)	C9—C14	1.386 (3)
N2—C8	1.344 (3)	C10—C11	1.378 (3)
N2—H2B	0.897 (10)	C10—H10	0.9300
N3—C1	1.376 (3)	C11—C12	1.384 (3)
N3—C17	1.441 (3)	C11—H11	0.9300
N3—C16	1.445 (3)	C12—C13	1.379 (4)
O1—C8	1.232 (3)	C12—C15	1.508 (3)
O2—C18	1.387 (3)	C13—C14	1.382 (3)
O2—H2	0.8200	C13—H13	0.9300
C1—C2	1.388 (3)	C14—H14	0.9300
C1—C6	1.402 (3)	C15—H15A	0.9600
C2—C3	1.374 (3)	C15—H15B	0.9600
C2—H2A	0.9300	C15—H15C	0.9600
C3—C4	1.381 (3)	C16—H16A	0.9600
C3—H3	0.9300	C16—H16B	0.9600
C4—C5	1.387 (3)	C16—H16C	0.9600
C4—C7	1.450 (3)	C17—H17A	0.9600
C5—C6	1.366 (3)	C17—H17B	0.9600
C5—H5	0.9300	C17—H17C	0.9600
C6—H6	0.9300	C18—H18A	0.9600
C7—H7	0.9300	C18—H18B	0.9600
C8—C9	1.485 (3)	C18—H18C	0.9600
			
C7—N1—N2	115.7 (2)	C9—C10—H10	119.7
C8—N2—N1	119.3 (2)	C10—C11—C12	121.4 (3)
C8—N2—H2B	121.7 (17)	C10—C11—H11	119.3
N1—N2—H2B	118.6 (17)	C12—C11—H11	119.3
C1—N3—C17	121.3 (2)	C13—C12—C11	117.7 (2)
C1—N3—C16	120.4 (2)	C13—C12—C15	121.2 (3)
C17—N3—C16	118.3 (2)	C11—C12—C15	121.1 (3)
C18—O2—H2	109.5	C12—C13—C14	121.3 (2)
N3—C1—C2	121.3 (2)	C12—C13—H13	119.4
N3—C1—C6	121.7 (2)	C14—C13—H13	119.4
C2—C1—C6	117.0 (2)	C13—C14—C9	120.7 (3)
C3—C2—C1	120.8 (2)	C13—C14—H14	119.6
C3—C2—H2A	119.6	C9—C14—H14	119.6
C1—C2—H2A	119.6	C12—C15—H15A	109.5
C2—C3—C4	122.3 (2)	C12—C15—H15B	109.5
C2—C3—H3	118.9	H15A—C15—H15B	109.5
C4—C3—H3	118.9	C12—C15—H15C	109.5
C3—C4—C5	116.9 (2)	H15A—C15—H15C	109.5
C3—C4—C7	120.0 (2)	H15B—C15—H15C	109.5
C5—C4—C7	123.0 (2)	N3—C16—H16A	109.5
C6—C5—C4	121.6 (2)	N3—C16—H16B	109.5
C6—C5—H5	119.2	H16A—C16—H16B	109.5
C4—C5—H5	119.2	N3—C16—H16C	109.5
C5—C6—C1	121.3 (2)	H16A—C16—H16C	109.5
C5—C6—H6	119.3	H16B—C16—H16C	109.5
C1—C6—H6	119.3	N3—C17—H17A	109.5
N1—C7—C4	122.5 (2)	N3—C17—H17B	109.5
N1—C7—H7	118.7	H17A—C17—H17B	109.5
C4—C7—H7	118.7	N3—C17—H17C	109.5
O1—C8—N2	122.2 (2)	H17A—C17—H17C	109.5
O1—C8—C9	121.1 (2)	H17B—C17—H17C	109.5
N2—C8—C9	116.8 (2)	O2—C18—H18A	109.5
C10—C9—C14	118.1 (2)	O2—C18—H18B	109.5
C10—C9—C8	123.2 (2)	H18A—C18—H18B	109.5
C14—C9—C8	118.6 (2)	O2—C18—H18C	109.5
C11—C10—C9	120.7 (2)	H18A—C18—H18C	109.5
C11—C10—H10	119.7	H18B—C18—H18C	109.5

### Hydrogen-bond geometry (Å, °)

**Table:**

*D*—H···*A*	*D*—H	H···*A*	*D*···*A*	*D*—H···*A*
N2—H2B···O2^i^	0.90 (1)	2.02 (1)	2.905 (3)	168 (2)
O2—H2···O1	0.82	1.92	2.720 (3)	166

Symmetry codes: (i) *x*−1, *y*, *z*.

## References

[bb1] Bruker (1998). *SMART*, *SAINT* and *SADABS* Bruker AXS Inc., Madison, Wisconsin, USA.

[bb2] Liu, H. (2010*a*). *Acta Cryst.* E**66**, o1582. 10.1107/S1600536810020763 PMC3006866 21587822

[bb3] Liu, H. (2010*b*). *Acta Cryst.* E**66**, o2026. 10.1107/S1600536810026723 PMC3007334 21588336

[bb4] Sheldrick, G. M. (2008). *Acta Cryst.* A**64**, 112–122. 10.1107/S0108767307043930 18156677

